# Fermented Herbal Formulas KIOM-MA128 Ameliorate IL-6-Induced Intestinal Barrier Dysfunction in Colon Cancer Cell Line

**DOI:** 10.1155/2016/6189590

**Published:** 2016-11-17

**Authors:** Kwang Il Park, Dong Gun Kim, Bo Hyoung Lee, Jin Yeul Ma

**Affiliations:** Korean Medicine (KM) Application Center, Korea Institute of Oriental Medicine (KIOM), 70 Cheomdan-ro, Dong-gu, Daegu 41062, Republic of Korea

## Abstract

Inflammatory bowel disease (IBD) comprises Crohn's disease (CD) and ulcerative colitis (UC). IBD increases the risk of colorectal cancer (CRC), depending on the extent and duration of intestinal inflammation. Increased IL-6 expression has been reported in IBD patients, which may be associated with intestinal barrier function through discontinuous tight junction (TJ). KIOM-MA is a specific agent for allergic diseases and cancer, and it is composed of several plants; these herbs have been used in traditional oriental medicine. We fermented KIOM-MA, the product of KIOM-MA128, using probiotics to improve the therapeutic efficacy via the absorption and bioavailability of the active ingredients. In this study, we demonstrated that KIOM-MA/MA128 exhibited anticolitis effects via the modulation of TJ protein. Interleukin-6 resulted in a dose-dependent decrease in the TER and an increase in the FITC-dextran permeability; however, pretreatment with 400 *µ*g/ml KIOM-MA/MA128 resulted in a significant increase in the TER and a decrease in the FITC-dextran permeability via IL-6 induction. Furthermore, protein and mRNA TJ levels remained stable after pretreatment with 400 *µ*g/ml KIOM-MA/MA128. Moreover, KIOM-MA/MA128 suppressed the expression of PLC*γ*1 and PKC. Taken together, these findings suggest novel information and clue of the anticolitis effects of KIOM-MA128 via regulation of tight junction.

## 1. Introduction

Inflammatory bowel disease (IBD) is composed of two major phenotypes, Crohn's disease (CD) and ulcerative colitis (UC). IBD affects more than 4 million individuals worldwide and frequently results in hospitalization or surgery; however, its molecular pathway remains unclear. The symptoms of IBD include abdominal pain, vomiting, diarrhea, rectal bleeding, and weight loss. Furthermore, IBD increases the risk of colorectal cancer (CRC), depending on the extent and duration of intestinal inflammation [1]. Several studies have suggested that a defective immune system and reduced intestinal function aggravate the pathogenesis of IBD [2].

Cytokines have been directly associated with the pathogenesis of IBD, and they play a critical role in the modulation of intestinal inflammation and the regulation of intestinal permeability [3]. Several lines of evidence suggest that cytokine increases disrupt intestinal barrier function. First, in vitro studies have indicated that IFN-*γ*, IL-1*β*, TNF-*α*, and IL-6 may cause barrier dysfunction in cultured epithelial monolayers [4–6]. In vivo, these cytokines may regulate disease severity and barrier function [7]. Specifically, experimentation with interleukin-6 has indicated that the regulation of tight junction proteins, such as ZO-1, ZO-2, and ZO-3, occludin, and claudins, increased the paracellular permeability via actin cytoskeleton modulation. Studies have suggested that a connection may exist between IBD and IL-6. IL-6 expression was increased in IBD patients [8], and this increased expression level may be related with intestinal barrier function through the discontinuous TJ and barrier dysfunction in inflammatory intestinal diseases. In addition, the downregulation of claudin-7 expression and the upregulation of claudin-2 may lead to modifications in the structures of TJs [9, 10]. In IL-6 (−/−) mice, the induction of acute colitis using DSS significantly inhibited the inflammatory response in the colon and decreased body weight loss compared with WT mice [11]. These findings illuminated the role of IL-6 signaling in IECs during DSS-induced inflammation and indicated that IL-6 is a key molecule in inflammatory bowel diseases.

In previous years, the treatment of IBD was based on nonspecific suppression of the immune response using corticosteroids or an immunosuppressive, such as azathioprine. Conventional treatment of colitis with an unspecific immune suppressor such as azathioprine may reduce duration of disease and help remission; however, there are severe side effects [12]. Many colitis drugs turn to unconventional treatments in the hopes of reducing the symptoms of disease, and about 40% of IBD patients use some form of mega-vitamin therapy, including herbal/dietary supplements.

Our group has indicated that KIOM-MA is a specific agent for allergic and chronic inflammatory diseases, and it is composed of several plants, such as* Glycyrrhizae radix, Polygoni cuspidati radix, Sophorae radix, Cnidii rhizoma, *and* Arctii fructus*; these herbs have been used in traditional oriental medicine in Asia [13, 14]. We fermented MA, the product of MA128, using probiotics to improve the therapeutic efficacy. The novel herbal medicine KIOM-MA increased the absorption and bioavailability of the active ingredients. Previous studies have demonstrated that MA128 possesses antiatopic dermatitis, antiasthma action, and anticancer [13, 15].

Despite many ongoing researches of integrative medicine and intestinal barrier function, the effect of herbal formula (KIOM-MA/MA128) is still unknown. In present study, we elucidate the effect of KIOM-MA/MA128 on cytokine-induced intestinal barrier dysfunction. Furthermore, we recently demonstrated that KIOM-MA/MA128 exhibited the inhibitory effect of intestinal barrier dysfunction via the regulation of TJ.

## 2. Materials and Methods

### 2.1. Cell Culture

Caco2 cells (ATCC, Manassas, VA, USA) were grown in EMEM media supplemented with 10% FBS, 1% antibiotic solutions (100,000 U/L penicillin and 100,000 mg/L streptomycin) at 37°C humidified atmosphere of 5% CO_2_. Culture medium was changed every 1-2 days. Upon approximately 90% confluence, cells were split using 0.25% trypsin. For growth on filters, Caco2 cells were plated on Transwell filters and measured TER. For experimental purposes, only Caco2 cells between passages 30 and 50 were used.

### 2.2. Reagents

IL-6 was procured from Sigma. Antibodies to occludin, ZO-1, claudin-2, MLC-2, and MLCK were purchased from Zymed Laboratories; the other antibodies were purchased from cell signaling. Alexa 594 secondary antibodies and DAPI (4#,6#-diamidino-2-phenylindole) were purchased from Molecular Probes. SYBR-Green PCR Master Mix is ABI (catalog number 43049155; Applied Biosystems). The standard of liquiritin was purchased from Sigma-Aldrich (St. Louis, MO, USA). Nodakenin, quercitrin, arctiin, matairesinol, and icariin were purchased from Chemfaces (Hubei, China). Arctigenin was purchased from MUST (Chengdu Must Bio-Technology Co., Ltd., Chengdu, China). Angoroside C, neolicuroside, (8S, 8′′R)-8-(4-hydroxy-3-methoxybenzyl)-8′′-(3′,4′-dimethoxybenzyl)-*γ*-butyrolactone 4-O-(*β*-D-glycopyranoside), and emodin 8-O-*β*-D-glucopyranoside 8 were obtained from Chungnam National University (Daejeon, Korea). The purity of the standards was all above 95%. HPLC grade acetonitrile was purchased from J. T. Baker Inc. (Philipsburg, NJ, USA) and acetic acid purchased from JUNSEI (Junsie Chemical Co., Ltd., Tokyo, Japan). Ultrapure water was prepared by Puris-Evo-UP Water System with Evo-UP Dio VFT and Evo-ROP Dico20 (Mirae ST Co., Ltd., Anyang, Gyeonggi-do, Korea). Ultrapure water (UW) was prepared with a resistivity of 18.2 MΩ cm^−1^ (Puris, Esse-UP Water System, Mirae ST Co., Anyang, Korea).

### 2.3. Preparation of the Fermented Herbal Cocktail MA128

The preparation of KIOM-MA128 has been previously described [15, 16]. Briefly, all herbs for the preparation of MA, including* Glycyrrhizae radix, Polygoni cuspidati radix, Sophorae radix, Cnidii rhizoma, *and* Arctii fructus*, were purchased from the Korea Medicinal Herbs Association (Yeongcheon, Korea). The identification of all herbs was confirmed by Professor KiHwan Bae of the College of Pharmacy, Chungnam National University (Daejeon, Korea), and all voucher specimens were deposited in the herbal band in the Korea Institute of Oriental Medicine (KIOM, Korea). MA was fermented at 37°C for 48 h using* L. rhamnosus* (1 × 10^8^ CFU/ml), followed by filtration through a 60 *μ*m nylon net filter (Millipore, Bedford, MA, USA). Three hundred seventy-six g of MA128 powder was produced, and the yield was 20.44%. The freeze-dried MA128 powder was dissolved in 10% (v/v) DMSO in DW. The solution was filtered (0.2 *μ*m, pore size) and maintained at 4°C prior to use.

*Chromatographic Conditions.* The KIOM-MA samples and standard samples were analyzed using Dionex HPLC System (Dionex Co., Sunnyvale, CA, USA), equipped with ultimate 3000 series binary pump, an autosampler, a column oven, and a diode array UV/VIS detector (DAD). Data analysis was performed by software named Dionex Chromelon. All chromatographic separations were performed on OptimaPak C18 column (5 *μ*m, 4.6 × 250 mm, RS Tech, Daejeon, Korea) and the column temperature was maintained at 40°C. The mobile phase was consisted of water with 0.2% acetic acid (A) and acetonitrile with 0.2% acetic acid (B). The gradient program was set as follows: 10–50% B at 0–60 min [13]. UV absorption was monitored at 203 nm and 280 nm at a flow rate of 1.0 ml/min. The total run time was 60 min, and the injection volume was 5 *μ*L.

### 2.4. Preparation of Standard Solutions and Samples

The stock solution of standards was prepared in 100% methanol at 1 mg/ml. KIOM-MA/MA128 samples were accurately weighted and diluted in methanol : DMSO (90 : 10, v/v). In all standards, KIOM-MA/MA128 were stored at 4°C. All working solutions were filtered through a 0.2 *μ*m syringe membrane filter from Whatman Ltd. (Maidstone, UK) before injection for HPLC analysis. 

*Cell Viability.* Cell viability was analyzed by Cell Counting Kit-8. This assay by utilizing Dojindo's highly water-soluble tetrazolium salt. Briefly, Caco2 cells (1 × 10^3^ cells) were plated on a 96-filter plate. After IL-6 or KIOM-MA, KIOM-MA128 treatment, add 10 *μ*l of the CCK-8 solution to each well of the plate, and incubate it for 1 hour at 37°C with 5% CO_2_. Absorbance was measured at 450 nm using a precision microplate reader (Molecular Devices, Sunnyvale, CA).

### 2.5. Measurement of Transepithelial Electrical Resistance (TER)

Caco2 cells were seeded at 1 × 10^5^cells in culture medium in 0.33 cm^2^ polyethylene terephthalate membrane inserts with 0.4 *μ*m pores (Millipore, Bedford, MA). The medium was changed every 2 days until complete differentiation. The electrical resistance was measured using a Millicell ERS-2 voltohmmeter (Millipore, Bedford, MA). The electrical resistance value was recorded for three consecutive measurements. Caco2 cells were treated with 50 ng/ml IL-6 or untreated following pretreatment with KIOM-MA/MA128, and the transepithelial electrical resistance (TER) was obtained at 18 and 24 h. The TER values were presented as Ohm cm^2^ and the experiments were conducted on 3 replicates from three independent experiments.

### 2.6. Epithelial Paracellular Permeability

The paracellular permeability was measured using a nonabsorbable, FITC-conjugated dextran probe (FD-4). Caco2 cells were plated at 1 × 10^5^cells in culture medium in 0.33 cm^2^ polyethylene terephthalate membrane inserts with 0.4 *µ*m pores (Millipore, Bedford, MA). Following pretreatment, the apical and basolateral sections were washed with PBS. FITC-dextran (1 mg/ml) was subsequently added to the apical side, and the basolateral side was added to PBS. Following 1 h of incubation at 37°C, 100 *µ*l of media from the basolateral side was plated to a 96-well plate, and the absorbance was measured by a precision microplate reader (Molecular Devices, Sunnyvale, CA). The excitation and emission wavelengths were 490 and 520 nm, respectively.

### 2.7. Immunofluorescent Staining for Junctional Proteins

Caco2 cells were seeded at 1 × 10^5^ cells on coverslips. On every 2 days, the medium was changed until full differentiation and cultured 24 hours following treatment with IL-6 and KIOM-MA/MA128. After treatment, the Caco2 cell monolayers were washed with the PBS (2 times), fixed using 4% formaldehyde for 10 min, and permeabilized (0.25% Triton X-100 in PBS) for 5 min at room temperature. The blocking was performed with 5% goat serum for 1 h at room temperature and incubated at 4°C overnight with primary antibody rabbit polyclonal anti-ZO-1 (1 : 50, diluted by 5% goat serum) and then washed with PBS, the incubation of secondary antibody for 1 h at room temperature (Alexa Fluor 594-anti-rabbit) and DAPI counterstaining. The TJ proteins images were obtained using a fluorescence microscope (OLYMPUS, Japan).

### 2.8. Western Blot

Western blot analysis was performed. Briefly, 5 × 10^5^ Caco2 cells per well were plated on 6 well plates. At the 24 h after treatment, Caco2 cell monolayers were washed with ice-cold PBS, and cells were lysed with radioimmunoprecipitation assay (RIPA) lysis buffer (Millipore Corporation, Billerica, MA, USA). The supernatant was collected, and amounts of protein were calculated using the BCA Protein Assay Kit. An equal amount of protein was separated on 10% SDS-PAGE gel. Proteins were transferred to PVDF (polyvinylidene fluoride) membrane (Millipore Corporation, Billerica, MA, USA). The membranes were blocked with 5% skimmed milk in TBS-T buffer for 1 h and then incubated with primary antibodies (anti-ZO-1 at 1 : 200, anti-occludin at 1 : 200, anti-claudin-2 at 1 : 200, anti-p-MLCK at 1 : 1000, anti-p-PKC*δ* at 1 : 1000, anti-p-MLC2 at 1 : 1000, and anti-tubulin at 1 : 2000) overnight at 4°C. After being washed in TBS-T buffer, the membranes were incubated with secondary antibodies for 1 h at room temperature. Protein bands were detected with Immobilon Western substrate (Millipore Corporation, Billerica, USA) and analyzed with the ChemiDoc Touch Imaging System (Bio-Rad, Hercules, CA, USA). The band density was normalized to the reference tubulin.

### 2.9. Quantitative Real-Time PCR

1 × 10^5^ Caco2 cells per well were plated in 6-well tissue culture-treated plates. At the end of the experiment, RNA was obtained using TRIzol reagent and chloroform. Reverse transcription was conducted in a 20 *μ*l reaction with 1 *μ*g of total RNA transformed into cDNA using AccuPower Cycle Script RT Premix (Bioneer). The measurements of ZO-1, ZO-2, and ZO-3, claudin-1, claudin-2, and claudin-7, occludin, and *β*-actin mRNA were conducted under the following conditions: 45 cycles of 95°C for 10 s, 60°C for 20 s, and 72°C for 30 s using a LightCycler 480 II (Roche, Rotkreuz, SWI). The mRNA level in each sample was quantified using the cycle threshold (Ct) value. The target genes were normalized relative to the reference gene actin.

### 2.10. Statistical Analysis

All statistical analyses were performed with SPSS version 18 and graphs were drawn with GraphPad Prism version 5. Experimental values are given as the means ± SEM. The statistical difference was determined by one-way ANOVA test. *p* values smaller than 0.05 were regarded as statistically significant.

## 3. Results

### 3.1. HPLC Analysis

**Figure 1:**
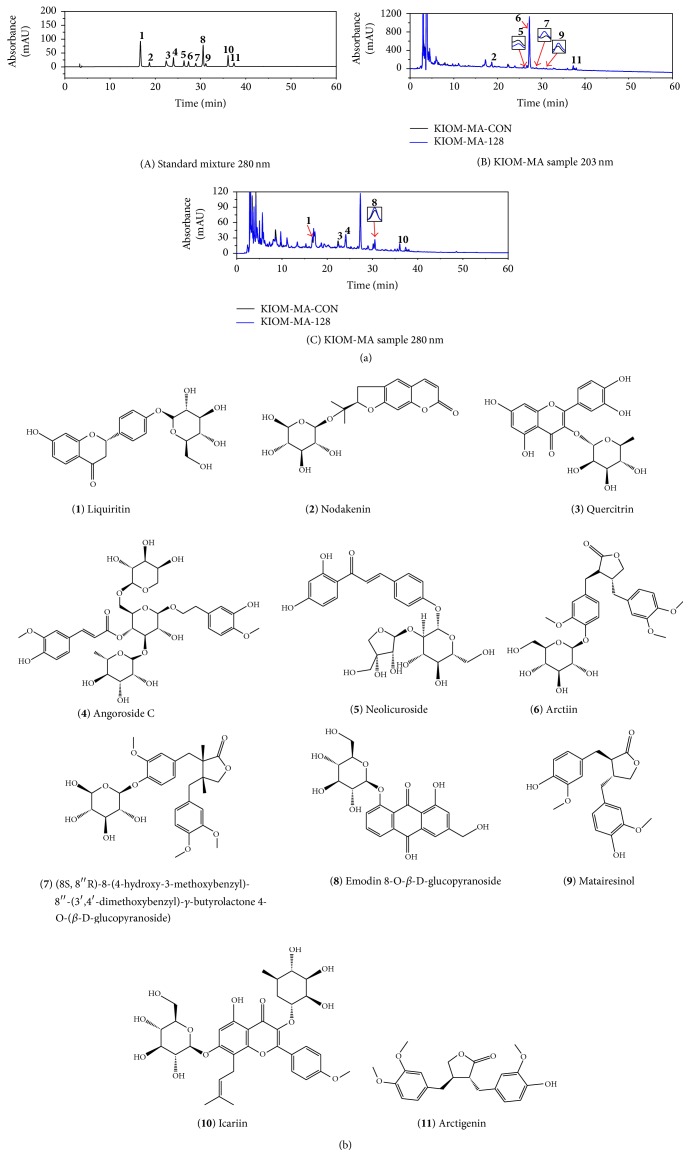
The HPLC chromatogram and the chemical structure of eleven markers of KIOM-MA/MA128. (a) The HPLC chromatogram of KIOM-MA/MA128. (b) The chemical structure of eleven markers of KIOM-MA/MA128.

Eleven marker compounds of KIOM-MA and KIOM-MA128 liquiritin (tR 16.7 min), nodakenin (tR 18.6 min), quercitrin (tR 22.3 min), angoroside C (tR 23.9 min), neolicuroside (tR 26.2 min), arctiin (tR 27.2 min), (8S, 8′′R)-8-(4-hydroxy-3-methoxybenzyl)-8′′-(3′,4′-dimethoxybenzyl)-*γ*-butyrolactone 4-O-(*β*-D-glucopyranoside) (tR 28.9 min), emodin 8-O-*β*-D-glucopyranoside (tR 30.5 min), matairesinol (tR 31 min), icariin (tR 36 min), and arctigenin (tR 37.2 min) were identified via HPLC analysis and compared with standard compounds (Figure 1(a), Table 1). Following fermentation, nodakenin (tR 18.6 min), quercitrin (tR 22.3 min), and neolicuroside (tR 26.2 min) were decreased, and the remaining compounds were increased in KIOM-MA128 (fermented KIOM-MA) following deglycosylation during fermentation. The structures of the KIOM-MA and KIOM-MA128 compounds are presented in Figure 1(b).

**Figure 2:**
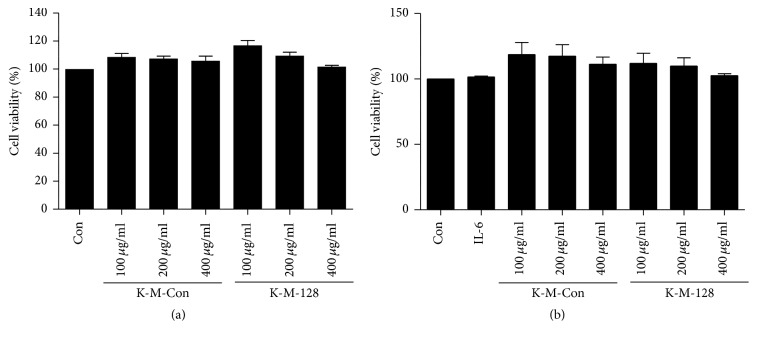
Effects of IL-6 and KIOM-MA/MA128 on cell viability of Caco2 cells. Cells were pretreated with various concentrations (100–400 *μ*g/ml) of KIOM-MA/MA128 for 1 h and treated with IL-6 (50 ng/ml) for 24 h. Cell viability was determined using a Cell Counting Kit-8. Cell viability is represented as the percentage relative absorbance compared with the controls. (a) Cell viability of KIOM-MA/MA128; (b) cell viability of KIOM-MA/MA128 with IL-6 (50 ng/ml). The results are represented as the means ± SDs of three independent experiments, and *t*-tests were performed.

*Effects of IL-6 and KIOM-MA/MA128 on Cell Viability.* We evaluated the effects of IL-6, KIOM-MA, and KIOM-MA128 on the cell viability in Caco2 monolayers. The Caco2 cell viability was measured following treatment with various concentrations of KIOM-MA/MA128 (100–400 *µ*g/ml) and incubation for 24 h. The findings indicated that KIOM-MA/MA128 with/without IL-6 did not affect the cell viability in the Caco2 monolayers (*p* > 0.05, Figure 2).

### 3.2. KIOM-MA/MA128 Prevented IL-6-Induced Epithelial Barrier Dysfunction in Caco2 Cell Monolayers

**Figure 3:**
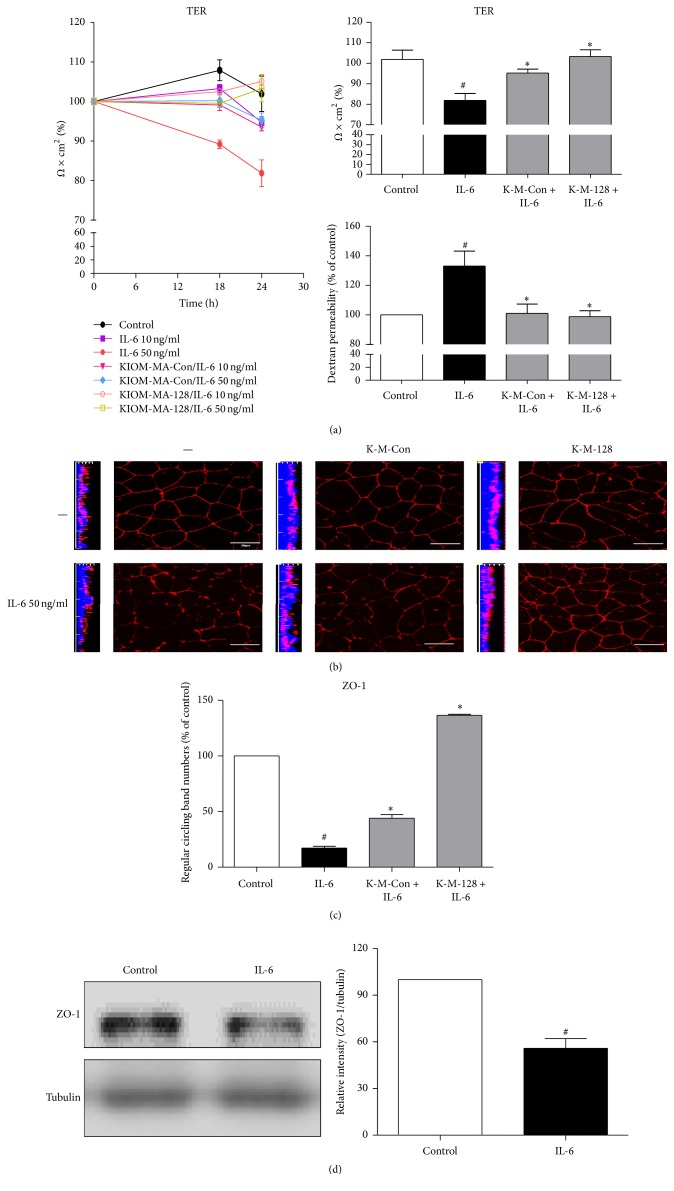
Effects of KIOM-MA/MA128 on Caco2 monolayer intestinal barrier function. (a) Intestinal barrier function was analyzed via the TER value, and paracellular permeability was determined using a nonabsorbable, fluorescein isothiocyanate- (FITC-) conjugated dextran probe (FD-30). Caco2 monolayer cells were pretreated with 400 *μ*g/ml KIOM-MA/MA128 1 h prior to IL-6 (10 ng/ml and 50 ng/ml) exposure for 24 h. (b) Caco2 monolayer cells were pretreated with 400 *μ*g/ml KIOM-MA/MA128 1 h prior to IL-6 (50 ng/ml) exposure for 24 h. ZO-1 was detected via immunofluorescence microscopy. Treatment of IL-6 caused significant disruptions of ZO-1. The ZO-1 staining was decreased in intensity and exhibited an irregular band. Four hundred *μ*g/ml KIOM-MA128 completely prevented ZO-1 in IL-6 treated Caco2 monolayers. (c) Histograms are represented by continuous bands encircling the cells at the cellular borders. (d) Cell lysates were analyzed for ZO-1 expression via Western blot. The results are reported as the means ± SDs from 3 independent experiments. ^*∗*^ *p* < 0.05 compared with the IL-6 treated group; ^#^ *p* < 0.05 compared with the control group.

Epithelial barrier dysfunction was measured as the flux of the FITC-dextran, and the TER was measured. Interleukin-6 resulted in a dose-dependent decrease in the TER (increase in the permeability) in the range of 10 and 50 ng/ml, with a maximal decrease in the TER at 24 h. Pretreatment with 400 *µ*g/ml of KIOM-MA and KIOM-MA128 resulted in a significant increase in the TER and a decrease in the FITC-dextran permeability via IL-6 induction. KIOM-MA128 exhibited a more substantial effect on the TER value and the FITC-dextran permeability compared with KIOM-MA (Figure 3(a)). The distribution of the protein ZO-1 was determined via an immunofluorescence assay using confocal microscopy (Figure 3(b)). ZO-1 was localized at the cell membrane as a continuous encircling band at the cellular borders in normal condition. The distribution of ZO-1 was disturbed and exhibited an irregular circling band at the cell membrane by IL-6 and pretreatment with KIOM-MA/MA128 prevented the disruption of ZO-1 in the Caco2 cell monolayer (Figure 3(c)). The expression level of ZO-1 was decreased in the IL-6-treated cells in cell lysate (Figure 3(d)). These morphological changes correlated with the results of the FITC-dextran permeability as manifested by the TER. Our findings also indicate that KIOM-MA128 may more effectively protect intestinal function via IL-6-mediated barrier dysregulation compared with KIOM-MA.

### 3.3. Effects of KIOM-MA/MA128 on IL-6-Induced Tight Junctional Protein Expression

**Figure 4:**
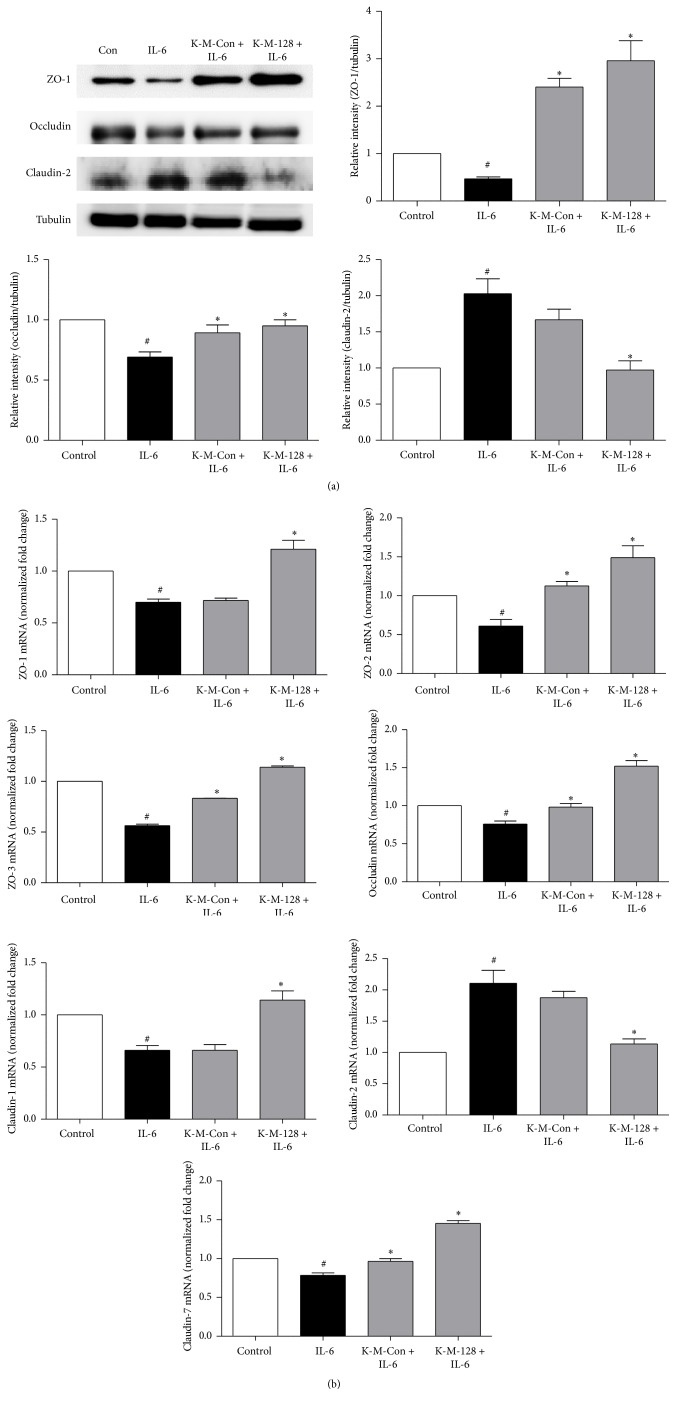
Effects of KIOM-MA/MA128 on the mRNA and protein expression of TJ proteins in the Caco2 monolayer. Caco2 monolayer cells were pretreated with 400 *μ*g/ml KIOM-MA/MA128 1 h prior to IL-6 (50 ng/ml) exposure for 24 h. (a) Cells were lysed, and the lysates were examined via Western blot for ZO-1, occludin, and claudin-2. Pretreatment with KIOM-MA/MA128 protected the TJ proteins. (b) Total mRNA was isolated, and the mRNA levels of TJ proteins, such as ZO-1, ZO-2, and ZO-3, occludin, and claudin-1, claudin-2, and claudin-7, were assessed. Pretreatment with KIOM-MA/MA128 protected the IL-6-induced mRNA expression. The results are reported as the means ± SDs from 3 independent experiments. ^*∗*^ *p* < 0.05 compared with the IL-6 treated group; ^#^ *p* < 0.05 compared with the control group.

To examine the effect of KIOM-MA/KIOM-MA128 on IL-6-induced TJ disruption, Caco2 cells were pretreated with 400 *µ*g/ml KIOM-MA/KIOM-MA128 for 1 h and incubated with IL-6 for 24 h. We analyzed the expression of tight junction proteins, including ZO-1, occludin, and claudin-2, via Western blot. The expression levels of occludin and ZO-1 were substantially decreased in the IL-6-treated cells. The cotreated KIOM-MA/MA128 cells exhibited an increased expression of ZO-1 and occludin. Moreover, the level of claudin-2 expression was significantly increased in the IL-6-treated cells. The cotreated KIOM-MA/MA128 cells exhibited an attenuated level of claudin-2 expression (Figure 4(a)). Our findings indicate that KIOM-MA/MA128 reduced the IL-6-induced disruption of TJ proteins, and KIOM-MA128 was more effective in providing protection compared with KIOM-MA.

### 3.4. Effects of KIOM-MA/MA128 on IL-6-Induced Tight Junctional mRNA Expression

We investigated the effects of KIOM-MA/MA128 on the mRNA levels of TJ proteins following IL-6 treatment using real-time PCR analysis. The Caco2 cells were pretreated with 400 *µ*g/ml KIOM-MA and KIOM-MA128 for 1 h and evaluated TJ-mediated mRNA, including ZO-1, ZO-2, and ZO-3 and occludin. The claudin family levels, such as claudin-1 and claudin-7, were decreased, whereas claudin-2 was increased in the IL-6-induced expression, and pretreatment of KIOM-MA/MA128 was downregulated. Furthermore, KIOM-MA128 demonstrated a more significant effect on TJ-mediated mRNA expression compared with the nonfermented KIM-MA (Figure 4(b)).

### 3.5. KIOM-MA128 Protected Caco2 Monolayer Barrier Function via Suppression of PLC*γ*1-PKC Pathway

**Figure 5:**
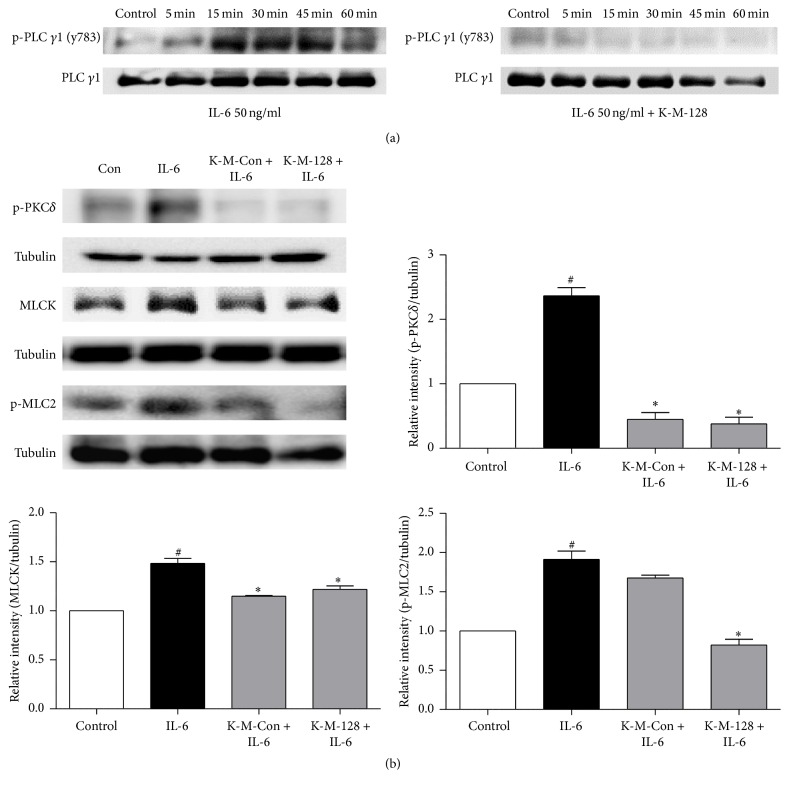
KIOM-MA/MA128 prevent the disruption of cytoskeleton by IL-6. (a) KIOM-MA/MA128 inhibited the IL-6-induced activation of PLC*γ*1. (b) Lysates were assayed for MLCK, MLC2, and PKC*δ* via Western blot. KIOM-MA/MA128 prevented the activation of MLCK, MLC2, and PKC*δ* induced by IL-6. The results are reported as the means ± SDs from 3 independent experiments. ^*∗*^ *p* < 0.05 compared with the IL-6 treated group; ^#^ *p* < 0.05 compared with the control group.

To examine the effect of KIOM-MA/MA128 on the cytoskeleton of Caco2 cells, a Western blot analysis was performed on cells treated with 400 *µ*g/ml KIOM-MA/MA128 for 24 h. As shown in Figure 5(a), the IL-6-treated cells increased the expression level of p-PLC*γ*1 (y783) at 30 min and decreased at 60 min. However, pretreatment with 400 *µ*g/ml KIOM-MA/MA128 for 1 h prior to IL-6 significantly decreased the IL-6-induced activation of p-PLC*γ*1 (y783). Furthermore, we assessed the MLCK pathway signaling to examine the epithelial barrier protection of KIOM-MA/MA128 by IL-6-induced epithelial barrier dysfunction. The pretreatment of Caco2 cells with KIOM-MA/MA128 for 1 h decreased the expression levels of p-PKC, MLCK, and p-MLC2 (Figure 5(b)). Furthermore, our findings indicate that KIOM-MA128 demonstrated a substantially stronger reducing effect on disruption of cytoskeleton by blocking the PLC*γ*1-PKC pathway compared with KIOM-MA.

## 4. Discussion

Many studies regarding natural herbal medicines have demonstrated the therapeutic potential of natural products in intestinal barrier function protection [17, 18]. Furthermore, our previous studies have demonstrated that KIOM-MA/MA18 have anti-inflammatory, anticancer, and antiatopic effects [13–15]. In this study, we analyzed the active ingredients of KIOM-MA/MA128 via HPLC and identified the altered eleven ingredients (Table 1).

Fermentation changes the decomposition of organic matter via microorganisms and produces numerous micromolecules from macromolecules. Many studies have demonstrated that fermentation by microorganisms improves the therapeutic efficacy, such as the absorption and bioavailability of the active ingredients [19–21]. Our findings demonstrated that arctigenin, arctiin, icariin, and matairesinol were increased, whereas nodakenin, neolicuroside, and quercitrin were decreased following fermentation (Figure 1). Arctigenin, arctiin, and matairesinol reinforced the intestinal barrier function through the regulation of paracellular permeability [22]. Moreover, icariin inhibited permeability in sertoli cells and the regulation of TJ proteins in pyramidal neurons [23, 24]. Here, we used KIOM-MA128, the fermentation of KIOM-MA, to determine whether it facilitated improvements in the protective effects on intestinal barrier dysfunction via the inhibition of actin cytoskeletal rearrangement.

The incidence rate of inflammatory bowel disease has continuously increased worldwide; however, its molecular pathway remains incompletely understood. Patients with Crohn's disease (CD) and ulcerative colitis (UC) exhibit increased intestinal permeability because of intestinal inflammation by external stimuli, such as pathogens, toxins, and other antigens. The entry of external pathogenesis induced the inflammatory response within the immune system, including mast cells and lymphocytes, and subsequently upregulated various cytokines, such as TNF-a, IL-1b, and IL-6. These cytokines increased the intestinal permeability through the downregulation of TER in intestinal epithelial cells. Our findings demonstrated that IL-6 disrupted the paracellular permeability and TER value; however, KIOM-MA/MA128 inhibited the penetration of FITC-dextran and reversed the decreasing TER value (Figure 3(a)).

TJs are not static barriers but are highly dynamic structures and cytoplasmic scaffolding proteins, such as ZO-1, ZO-2, and ZO-3, that act to anchor the actin cytoskeleton [25], which plays a critical role in TJ structure and function. These TJ proteins modulate homeostasis in the intestinal tract through the regulation of nutrients, ions, and water entry. IL-6 was increased in IBD patients and modulated the expression of ZO-1 and claudin-2 proteins in vivo and in vitro. This study indicated the anticolitis effect of KIOM-MA/MA128 using IL-6-mediated intestinal barrier dysfunction in the Caco2 cell monolayer. Our findings demonstrated that KIOM-MA/MA128 increased the mRNA levels of ZO-1, ZO-2, and ZO-3, occludin, and claudin-1 and claudin-7 and decreased the mRNA level of claudin-2 following IL-6-induced dysregulation of the mRNA levels of TJ proteins (Figure 4(b)). Furthermore, IL-6 treated cells downregulated the ZO-1 and occludin expression; however, pretreatment with KIOM-MA/MA128 protected these proteins (Figure 4(a)).

Cytokine-induced changes in paracellular permeability contribute to diverse pathological and physiological conditions, which are caused by the regulation of intestinal barrier integrity. Cytokines, including IFN-*γ*, TNF-*α*, and interleukins, affect the actin-myosin cytoskeleton linked by scaffolding proteins [26]. In TJ regulation signaling pathways, the relationships between TJ proteins and the actomyosin ring are regulated by diverse signaling proteins, such as mitogen-activated protein kinases (MAPK), protein kinase C (PKC), myosin light chain kinase (MLCK), and the GTPases. Furthermore, PLC*γ*1 plays a role in actin reorganization via the inhibition of PKC pathway and Ca^2+^ signaling [27–29]. PKC isoforms have also been indicated to modulate the TER value in intestinal epithelial cell monolayers [30]. The activations of PKC*α* and PKC*δ* lead to the activation of MLCK and increase the TER value, paracellular permeability, and redistribution of ZO-1. The phosphorylation of myosin II regulatory light chain (MLC) by MLCK plays a pivotal role in contractions in the actomyosin ring. The inhibition of MLCK prevents increases in TJ permeability [31]. MLCK-mediated regulation of TJ permeability by diverse extracellular stimuli, such as cytokines and bacteria, is a critical pathway to maintain the TJ barrier [32, 33]. The phosphorylation of MLC is also associated with the assembly and disassembly of TJs. In a recent study, the upregulation of MLCK expression in differentiated Caco2 cell monolayers led to a reduction in the TER and redistribution of ZO-1 and occludin [34, 35]. Our data demonstrated that IL-6 increased the paracellular permeability through the activation of PKC*δ*, MLCK, and p-MLC2 via the activation of PLC*γ*1; however, KIOM-MA/MA128 inhibited the activation of PLC*γ*1, PKC*δ*, MLCK, and p-MLC2 in the Caco2 cell monolayer (Figures 5(a) and 5(b)).

## 5. Conclusions

These findings provide crucial results that KIOM-MA/MA128 prevented IL-6-induced colitis through the regulation of TJ proteins, and these findings were correlated with MLCK inhibition. Additional investigations are necessary to understand the molecular mechanism of KIOM-MA/MA128 in the interrelation with intestinal barrier function. In conclusion, our study suggests that KIOM-MA and KIOM-MA128 protect the IL-6-induced TJ dysfunction in the Caco2 cell monolayer through the upregulation of ZO proteins (ZO-1, ZO-2, and ZO-3), occludin, and claudin-1 and claudin-7, as well as the downregulation of claudin-2, p-PKC*δ*, MLCK, and p-MLC2 via a PLC*γ*1-dependent pathway. These findings provide alternative targets in the treatment of IBD, and KIOM-MA128 is more effective for the treatment of IBD compared with KIOM-MA. Finally, the current findings provide support for the anticolitis effects of KIOM-MA128 in regard to pharmacological evidence.

## Figures and Tables

**Table 1:** List of the changed eleven markers and peak areas from KIOM-MA/MA128.

Number	Compound name	KIOM-MA area (mAU)	KIOM-MA128 area (mAU)
**1**	Liquiritin	4.41	4.57
**2**	Nodakenin	16.35	14.84
**3**	Quercitrin	22.77	13.93
**4**	Angoroside C	3.89	6.48
**5**	Neolicuroside	0.31	0.21
**6**	Arctiin	233.51	246.11
**7**	(8S, 8′′R)-8-(4-hydroxy-3-methoxybenzyl)-8′′-(3′,4′-dimethoxybenzyl)-*γ*-butyrolactone 4-O-(*β*-D-glucopyranoside)	0	2.24
**8**	Emodin 8-O-*β*-D-glucopyranoside	2.66	2.57
**9**	Matairesinol	1.37	3.19
**10**	Icariin	5.64	6.37
**11**	Arctigenin	3.8	14.34
